# Genomic evidence links human dengue cases with undetermined serotypes to sylvatic lineages

**DOI:** 10.1186/s41182-025-00795-5

**Published:** 2025-08-25

**Authors:** Jeyanthi Suppiah, Murni Maya Sari Zulkifli, Amir Hussin Adiee, Nur Ain Zahidah Zainudin, Mazrul Effendy Dukut Soeharto, Nuraisyah Ramli, Ming Keong Wan, Mohd Rahim Sulong, Zailiza Suli, Rozainanee Mohd Zain

**Affiliations:** 1 Virology Unit, Infectious Disease Research Centre, Institute for Medical Research, National Institutes of Health, Ministry of Health, Shah Alam, Selangor Malaysia; 2 Vector-Borne Disease Sector, Disease Control Division, Ministry of Health, Putrajaya, Malaysia

**Keywords:** Sylvatic dengue, RT-PCR, Mutation, Phylogenetic, Metagenomics

## Abstract

**Background:**

Sylvatic dengue viruses, typically maintained in non-human primate and forest mosquito cycles, have rarely been associated with human infections. However, sporadic spillovers have been reported in Southeast Asia, including Malaysia. These events are often under-detected due to the genetic divergence of sylvatic strains from endemic urban dengue viruses. During routine surveillance in Malaysia (2024–2025), a subset of clinically confirmed dengue cases yielded undetectable serotype results by commercial real-time reverse transcription polymerase chain reaction (RT-PCR) assays, prompting investigation into a possible sylvatic origin.

**Methods:**

We investigated 22 such cases through clinical, serological, molecular, and phylogenetic analyses. NS1 antigen and broad-range RT-PCR confirmed acute dengue infection. Selected samples underwent sequencing and lineage determination.

**Results:**

Most patients presented with severe dengue during early illness (mean day 3), with 95.5% NS1 positivity and predominantly primary infection profiles. Despite serotyping failure, sequencing revealed that eight of nine analyzed samples belonged to sylvatic DENV2, while one represented a divergent DENV3. Comparative amino acid analysis uncovered a unique signature in recent Malaysian sylvatic DENV2 strains, differentiating them from both urban and historical sylvatic lineages. This includes the V270 mutation in the M gene; R844, V884, and I898 in the NS1 gene; T1207 in the NS2A gene; A1597 in the NS3 gene; and D3048 and I3373 in the NS5 gene. Phylogenetic analysis clustered these strains into a distinct Malaysian clade, separate from the African sylvatic lineage.

**Conclusions:**

This study provides the first genomic evidence of a recent sylvatic DENV2 spillover into humans in Malaysia, likely undetected by standard diagnostics due to genetic divergence. These findings underscore the urgent need to enhance surveillance tools and explore the sylvatic transmission cycle’s role in dengue epidemiology.

## Introduction

Sylvatic dengue represents a distinct ecological form of the dengue virus (DENV), maintained predominantly in transmission cycles between non-human primates and forest-dwelling mosquito species. Unlike the urban transmission cycle, which involves sustained human-to-human spread primarily through *Aedes aegypti* and *Aedes albopictus*, sylvatic dengue persists in enzootic cycles facilitated by forest-adapted vectors, such as *Aedes furcifer*, *Aedes luteocephalus*, and *Aedes niveus* [1]. Phylogenetic analyses indicate that contemporary urban DENV lineages likely originated from these sylvatic ancestors, which gradually adapted to anthropophilic transmission pathways over time [2, 3]. Notably, sylvatic strains have been identified for at least three serotypes; DENV1, DENV2, and DENV4. [4].

Although sylvatic dengue viruses are largely confined to these wildlife cycles, they possess the capacity to spill over into human populations as documented in Table 1. Such spillover events are increasingly plausible in the context of anthropogenic disturbances, particularly deforestation, agricultural expansion, and urbanization that bring humans into closer contact with sylvatic habitats [5]. This raises important concerns regarding the potential for sylvatic DENV strains to adapt to human hosts and vectors, thereby establishing new urban transmission cycles and contributing novel genetic diversity to the global dengue virus pool. Despite these risks, sylvatic dengue is not routinely screened in clinical and epidemiological surveillance due to its rarity in human cases. Standard molecular diagnostic assays are primarily designed to detect urban DENV genotypes, increasing the likelihood that sylvatic infections are either missed or misclassified. Diagnostic challenges are further compounded by serological cross-reactivity between sylvatic and urban strains, which limits the specificity of widely used serological tests. In addition, vector surveillance programs seldom include forest-associated mosquito species, resulting in limited data on the prevalence, distribution, and vector competence of sylvatic vectors.

**Table 1:** Documented cases of sylvatic dengue infection in human population

No	Virus strain	DENV type	Infected host	Country	Years	Clinical classification (WHO 2009)	Reference
1	36046/05	Sylvatic DENV1	Human	Malaysia	2005	Dengue with warning signs	[7]
2	DakAr-HD10674	Sylvatic DENV2	Human	Senegal	1970	Unknown	[6]
3	IBH11208/IBH11234/IBH11664	Sylvatic DENV2	Human	Nigeria	1966	Dengue without warning signs	[6]
4	DKD811	Sylvatic DENV2	Human	Malaysia	2008	Severe dengue	[8]
5	EEB-17	Sylvatic DENV2	Human	Spain	2009	Severe dengue	[11]
6	D2Sab2015	Sylvatic DENV2	Human	Malaysia	2015	Unknown	[12]
7	DKE121	Sylvatic DENV4	Human	Malaysia	2007	Unknown	[13]

A handful of studies have documented the presence of sylvatic dengue virus in Malaysia across multiple hosts, including the human population [6–8], sentinel silver leaf monkeys [9], and urban mosquito populations [10]. These findings suggest that Malaysia may act as a natural reservoir for a diverse array of sylvatic dengue strains. However, much of the existing evidence stems from historical investigations, with recent data remaining scarce and under-represented. In light of this gap, this study was conducted to explore the genomic signatures of recent sylvatic dengue cases that were unexpectedly identified among clinical samples submitted to our laboratory in Malaysia for routine dengue molecular testing.

## Materials and methods

### Study design and setting

This study was undertaken by the Virology Laboratory at the Institute for Medical Research, National Institutes of Health, Malaysia, utilizing archived clinical specimens (serum or plasma) originally submitted for routine dengue virus serotyping between January 2024 and March 2025.

### Antigen and serology tests

Antigen and serological testing of DENV infection was carried out by the respective hospitals or health clinics at the time of patient admission, prior to the submission of samples to our laboratory. This test was performed using the approved dengue rapid test kit at their facility, which detects NS1 antigen and IgM and IgG antibodies. The test results were duly recorded on the accompanying patient request forms submitted alongside the clinical specimens.

### Viral RNA extraction and DENV serotyping

Viral RNA was extracted from 140 μl of patient serum/plasma samples using the QIAamp Mini Viral RNA Extraction Kit (Qiagen, USA). Dengue virus detection and serotyping were carried out on NS1-positive cases or cases with unknown causes of death, and in selected instances to rule out dengue infection. A commercial real-time RT-PCR dengue serotyping kit was employed as the primary diagnostic method alongside a universal single probe pan-dengue real-time RT-PCR assay [14], designed for broad dengue virus detection without serotyping, as a supplementary test. Ambiguous results were further confirmed through sequencing.

### Viral RNA purification

Prior to sequencing, 10 µl of extracted viral RNA was subjected to ribosomal RNA (rRNA) depletion, the initial step in the TruSeq Stranded Total RNA Kit protocol (Illumina, USA). This was followed by RNA purification using the sodium acetate–glycogen precipitation method. The RNA was first diluted to a final volume of 180 µL with nuclease-free water, then mixed with 18 µL of sodium acetate (Nacalai Tesque, Japan) and 1.5 µl of 20 mg/mL glycogen (Thermo Scientific, USA). Subsequently, 600 µl of absolute ethanol (Sigma, USA) was added, and the mixture was vortexed thoroughly. The solution was incubated at −20 °C for 2 h to promote RNA precipitation. Following incubation, the sample was centrifuged at 10,000×*g* for 30 min at 4 °C. The supernatant was carefully removed, and the resulting RNA pellet was washed once with 200 µl of 70% ethanol and centrifuged again at 4 °C for 5 min. The supernatant was discarded, and the pellet was air-dried for 5 min before being resuspended in 8.5 µl of elution buffer.

### Metagenomic sequencing

Full-genome sequencing by metagenomic approach was performed on presumptive dengue cases, with a cycle threshold (Ct) of less than 30. DNA library was prepared from the purified RNA using the TruSeq Stranded Total RNA kit (Illumina, USA), following the manufacturer's protocol. Key steps included first and second-strand cDNA synthesis, adenylation, adapter ligation, and library amplification. Sequencing was performed on an Illumina NextSeq 500 system, with a final loading concentration of 1.2 pM applied for a mid-output configuration.

### Genome data and phylogenetic analysis

High-quality sequencing reads were generated through trimming and filtering using BBDuk (BBTools v38.57), followed by de novo assembly with MEGAHIT v1.2.8. DENV-specific contigs were identified via sequence similarity searches using BLASTN (v2.9.0+), and full-genome sequences were extracted in FASTA format. Reference sylvatic dengue virus genomes were retrieved from the National Center for Biotechnology Information (NCBI). Multiple sequence alignment of the complete genomes was conducted using MEGA-X, allowing for the identification of unique mutations. Phylogenetic analysis was carried out using the neighbor-joining method, and pairwise genetic distance calculations were performed to assess genetic diversity across clades and sub-clades.

## Results

### Characteristics of serotype-undetermined dengue cases

A total of 22 dengue cases from 2024 to 2025 were initially undetected by a commercial RT-PCR serotyping kit but were subsequently picked up by a pan-dengue detection assay performed in parallel. Case characteristics are shown in Table 2. Among these, patient ages ranged from 11 to 80 years, with a mean age of approximately 41 years. The majority of patients were male (*n* = 15, 68.2%), while the remaining seven cases (31.8%) were female.

**Table 2:** Demography, clinical and laboratory details of serotype-undetermined dengue cases from years 2024 to 2025

No	Lab ID	Locality	Age/gender	Clinical diagnosis	Sample received	Commercial kit result	Broad detection assay (Ct)
1	UDS24/7	Sarawak	80/M	Fatal severe dengue with occult bleeding and multiorgan failure (day 3)	15.03.2024	Not detected	Detected (Ct: 28.00)
2	UDS29/8	Sarawak	38/M	Severe dengue in compensated shock (day 5)	04.04.2024	Not detected	Detected (Ct: 32.00)
3	UDS37/9	Sarawak	31/F	Severe dengue with plasma leakage (day 2)	21.05.2024	Not detected	Detected (Ct: 26.00)
4	UDS47/9	Sarawak	45/F	Severe dengue (day 4)	05.07.2024	Not detected	Detected (Ct: 32.00)
5	UDS70/2	Kelantan	16/M	Severe dengue (day 3)	27.09.2024	Not detected	Detected (Ct: 23.60)
6	UDS70/3	Kelantan	58/F	Severe dengue (day 3)	27.09.2024	Not detected	Detected (Ct: 27.80)
7	UDS70/5	Kelantan	31/F	Severe dengue with transaminitis (day 3)	27.09.2024	Not detected	Detected (Ct: 24.90)
8	UDS71/6	Perak	31/M	Dengue with warning sign and resolved shock (day 5)	03.10.2024	Not detected	Detected (Ct: 34.60)
9	UDS73/8	Kelantan	55/F	Severe dengue in compensated shock (day 5)	07.10.2024	Not detected	Detected (Ct: 26.80)
10	UDS74/3	Kelantan	16/M	Severe dengue with plasma leakage (day 3)	09.10.2024	Not detected	Detected (Ct: 32.70)
11	UDS74/6	Kelantan	68/M	Severe dengue with carditis (day 1)	09.10.2024	Not detected	Detected (Ct: 27.30)
12	UDS7/50	Kelantan	53/M	Severe dengue with decompensated shock and transaminitis	09.10.2024	Not detected	Detected (Ct: 31.20)
13	UDS7/70	Kelantan	26/M	Severe dengue (day 3)	15.10.2024	Not detected	Detected (Ct: 30.40)
14	UDS80/6	Kelantan	70/M	Severe dengue with resolved decompensated shock (day 4)	23.10.2024	Not detected	Detected (Ct: 33.60)
15	UDS8/60	Kelantan	55/M	Dengue fever (day 4)	06.11.2024	Not detected	Detected (Ct: 32.70)
16	UDS89/2	Kelantan	67/M	Severe dengue with transaminitis	14.11.2024	Not detected	Detected (Ct: 35.90)
17	UDS90/5	Kelantan	45/M	Severe dengue with transaminitis (day 7)	18.11.2024	Not detected	Detected (Ct: 34.40)
18	UDS93/1	Sarawak	48/F	Severe dengue with pleural effusion and transaminitis (day 4)	21.11.2024	Not detected	Detected (Ct: 26.50)
19	UDS98/2	Kelantan	26/M	Severe dengue with plasma leakage (day 4)	04.12.2024	Not detected	Detected (Ct: 37.29)
20	UDS103/1	Kelantan	15/M	Severe dengue (day 5)	19.12.2024	Not detected	Detected (Ct: 28.00)
21	UDS29/4	Kelantan	11/F	Dengue fever, no warning signs	26.03.2025	Not detected	Detected (Ct: 32.11)
22	UDS48/8	Pahang	26/M	Severe dengue (day 5)	30.03.2025	Not detected	Detected (Ct: 32.00)

These cases were geographically distributed across several Malaysian states, with Kelantan accounting for the highest number (*n* = 15, 68.2%), followed by Sarawak (*n* = 5, 22.7%), and one case each from Perak and Pahang (4.5% each). The highest concentration of cases within Kelantan came from the districts of Jeli, Tanah Merah, and Kuala Krai.

In terms of clinical presentation, 19 of the 22 patients (86.4%) were diagnosed with severe dengue, including complications, such as plasma leakage, transaminitis, occult bleeding, carditis, and shock. Two patients (9.1%) presented with uncomplicated dengue fever, and one case (4.5%) involved dengue with warning signs and resolved shock.

The Ct values for these positive detections ranged from 23.60 to 37.29, with an average Ct value of approximately 30.30, suggesting a generally moderate to low viral load at the time of testing. Most samples were collected during the early phase of illness, between onset days 1–7, with day 3 being the most frequently recorded (*n* = 6).

### Antigen and serological profile

NS1 antigen testing was positive in 21 out of the 22 cases (95.5%), indicating a strong presence of early dengue viral antigen across the cohort. One case (UDS8/60) had an unknown NS1 status. IgM status was reported as negative in 14 cases, positive in 2 cases, and unknown in 6 cases. IgG was negative in 12 cases, positive in 5, and unknown in 5 cases. Among the cases with available IgM and IgG results, the majority (12/16) demonstrated a primary infection profile**,** indicated by NS1 positivity with both IgM and IgG negative. A smaller subset (4/16) showed evidence of secondary infection, such as UDS70/3, UDS71/6, UDS80/6 and UDS89/2.

### Genomic profile

Sequencing was successfully performed on nine selected serotype-undetermined dengue cases, yielding either full or partial genome data (Table 3). Of these, five cases (UDS70/2, UDS70/5, UDS73/8, UDS74/6, and UDS93/1) yielded full-genome sequences**,** while four others (UDS37/9, UDS70/3, UDS7/70 and UDS103/1) generated partial sequences ranging from 214 to 3427 base pairs.

**Table 3:** Sequencing and lineage identity of selected cases with unresolved dengue serotype

No	GISAID Accession No	ID	Sequencing status	Lineage identity
1	EPI_ISL_20063516	UDS37/9	Partial C/M/NS1/NS5, 3427 bp	Sylvatic DENV2
2	EPI_ISL_20063517	UDS70/2	Full genome	Sylvatic DENV2
3	EPI_ISL_20063518	UDS70/3	Partial C/pre-M, 498 bp	Sylvatic DENV2
4	EPI_ISL_20063519	UDS70/5	Full genome	Sylvatic DENV2
5	EPI_ISL_20063520	UDS73/8	Full genome	Sylvatic DENV2
6	EPI_ISL_20063521	UDS74/6	Full genome	Sylvatic DENV2
7	EPI_ISL_20063522	UDS7/70	Partial NS5, 214 bp	Sylvatic DENV2
8	EPI_ISL_20063523	UDS93/1	Full genome	Divergent DENV3
9	EPI_ISL_20064972	UDS103/1	Partial C/pre-M, 370 bp	Sylvatic DENV2

Lineage analysis revealed that eight of the nine sequences were classified as sylvatic DENV2, including both full genomes and partial sequences. Notably, one case (UDS93/1) was identified as a divergent DENV3, based on the phylogenetic tree, indicating the potential emergence or spillover of non-typical DENV lineages.

### Amino acid variability analysis

**Fig. 1:**
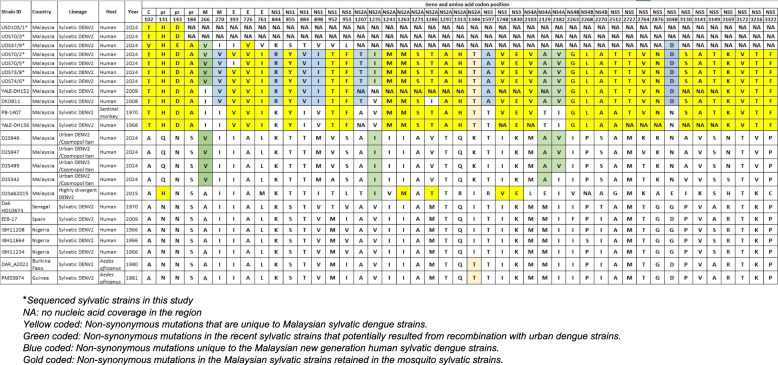
Amino acid variability in Malaysian human sylvatic DENV2 strains compared to strains from other regions and sources

A comparative analysis of amino acid sequences from Malaysian sylvatic DENV2 strains infecting humans, isolated in 2024, revealed a highly conserved and distinct mutational profile across multiple viral proteins (Fig. 1). These recent strains, with the exception of a divergent UDS37/9 strain, exhibited a consistent pattern of amino acid substitutions in structural and non-structural proteins, which were also present in the older 2008-DKD811 and 2009-YALE-DH152 strains but absent in both the historical sylvatic strains (1968-YALE-DH136 and 1970-P8-1407) and urban DENV2 strains from Malaysia. This subset of amino acids (highlighted in blue in Fig. 1), V270 in the M gene; R844, V884, and I898 in the NS1 gene; T1207 in the NS2A gene; A1597 in the NS3 gene; and D3048 and I3373 in the NS5 gene, may serve as additional molecular markers to distinguish the recent emerging lineage from the historical sylvatic and endemic strains.

**Fig. 2:**
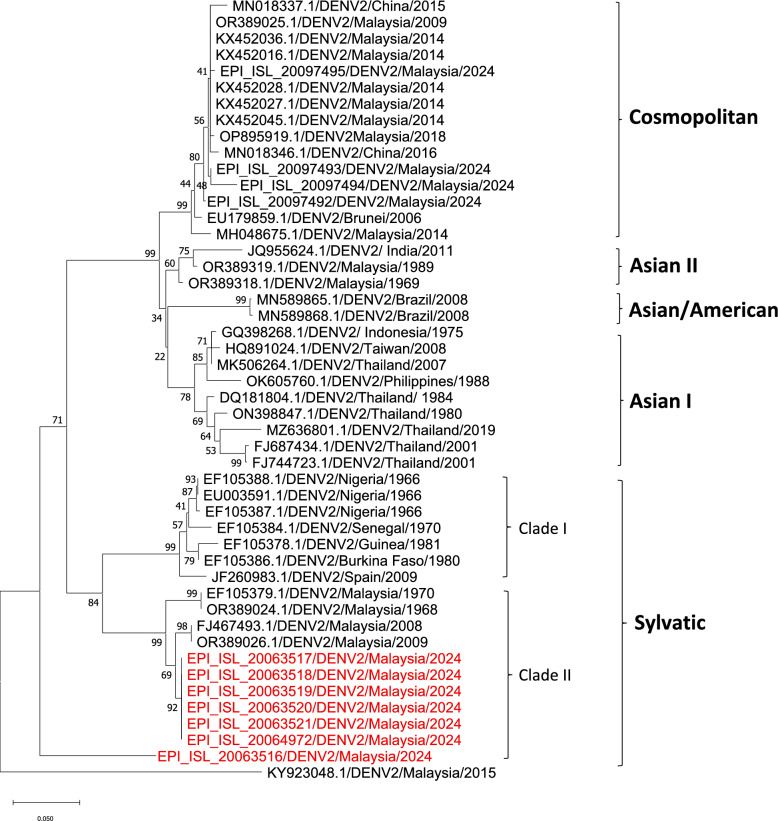
Phylogenetic tree of DENV2 clustered the sequences from this study (*red*) into sylvatic dengue lineage. UDS70/7 was excluded from this analysis due to the generation of very small contig during sequencing. Nevertheless, BLAST results indicated strong similarity of this strain to sylvatic DENV2

Several substitutions appear to be uniquely conserved among the Malaysian sylvatic strains (both historical and recent), further underscoring the molecular distinctiveness of the Malaysian sylvatic lineage. These mutations (highlighted in yellow) clearly differentiate the Malaysian sylvatic strains from those of other regions and are associated with the formation of Clade II. Phylogenetic analysis (Fig. 2) also supports this divergence, clearly resolving the sylvatic DENV2 sequences into two separate clades: Clade I, comprising African-origin sylvatic strains, and Clade II, encompassing the Malaysian sylvatic strains.

In contrast, a few amino acid mutations were found to be conserved between the 2024 sylvatic DENV2 strains infecting humans and known urban DENV2 strains isolated within the same year, but notably absent in older sylvatic lineages. These shared mutations include V266 in the M gene, I1235 in NS2A, and both A2129 and V2182 in the NS4A gene region.

**Fig. 3:**
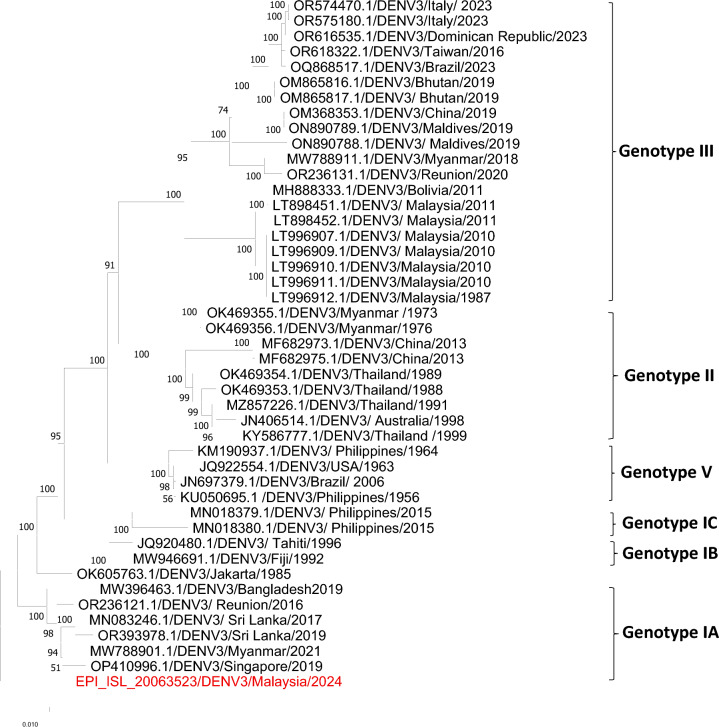
Phylogenetic tree of DENV3 clustered one sequence from this study (*red*) into a divergent DENV3 lineage

Interestingly, one isolate (UDS93/1) was identified as a divergent DENV3 strain based on the phylogeny (Fig. 3). Although the Nextclade tool classified this strain as genotype 1a, the presence of several unique mutations underscores its genetic diversity. This includes S357 in the C gene, G442 in prM, G1247 and R1877 in the E gene. Additional there was insertion of GEKKLR at codon position 1996 in the E gene.

## Discussion

The Virology Unit, Institute for Medical Research, serves as one of the national reference laboratories for dengue PCR serotyping in Malaysia. From 2024-2025, 22 cases of unknown serotype were identified among routine PCR test samples submitted for diagnostic confirmation. To meet reporting obligations and confirm these atypical findings, full-genome sequencing was initiated. Of these, eight samples yielded partial to complete genomes, revealed as sylvatic DENV2, while one sample was identified as a divergent DENV3. Low viral load in the remaining samples limited sequencing success, precluding full lineage assignment. Nonetheless, their strong resemblance in geographic location and sampling date to the confirmed sylvatic cases suggests a likely association. The detection of sylvatic DENV2 in multiple unrelated cases across different districts in Malaysia supports the possibility of low-level sylvatic transmission to humans, potentially contributing to the failure of commercial serotyping assays to detect these strains. These findings highlight the importance of genomic surveillance, especially for atypical or genetically distinct dengue strains.

Although human infection by sylvatic dengue viruses is uncommon, it has been reported sporadically. In Malaysia, an ancestral sylvatic DENV1 strain was previously isolated from a human case, sharing over 97% genomic similarity with a DENV1 strain from a sentinel monkey captured in 1972 [7] Globally, the first human case of sylvatic DENV2 was recorded in Nigeria in 1966 [15] followed by a similar case in Senegal in 1970 [16]. A 2020 outbreak in West Africa further suggests that sylvatic infections remain under-recognized due to diagnostic limitations [17]. A 2008 study described the isolation of a sylvatic dengue virus type 2 (DENV2) from a human patient in Malaysia. The complete genome sequence of this virus closely matched that of a sylvatic DENV2 strain isolated from a monkey in the same region in 1970 [8]. This finding marked the first isolation of a sylvatic dengue virus in Asia in over three decades.

Sylvatic DENV4 has been identified in several regions, notably in Malaysia and West Africa. It has been detected in *Aedes niveus* mosquitoes collected from the forest canopy, suggesting their role as vectors in the sylvatic cycle [18]. In terms of human infection, a novel DENV4 strain, DKE-121 was isolated from a hospitalized 37-year-old farmer in Malaysia and sequencing revealed that it is more related to sylvatic DENV4 [13].

In this study, phylogenetic analysis delineated two distinct sylvatic DENV2 clades: Clade I (African origin) and Clade II (Malaysian origin). The 1960s and 1970s Malaysian sylvatic strains are considered baseline references for the classical sylvatic DENV2 genotype, before major anthropogenic changes and inter-ecological virus mixing. The 2008–2009 isolates represent more recent spillover events but still pre-date the 2024 emergence and lack the mutations shared with urban strains. This consistent mutational pattern suggests strong intra-lineage conservation and likely reflects a localized and recent zoonotic emergence of this sylvatic DENV2 variant in human populations. This phylogeographic clustering further reinforces the distinct evolutionary trajectory and possible endemic expansion of Clade II within Malaysia.

Based on the amino acid variability analysis of sylvatic DENV2, three distinct categories of mutations were identified. The first category comprised mutations that were exclusively conserved across all Malaysian sylvatic strains, both historical and recent, highlighting lineage-specific signatures. The second category included mutations uniquely present in the recent Malaysian sylvatic strains isolated from human infections, distinguishing from historical strains and suggesting possible adaptation or diversification.

The third category consisted of mutations that were shared between the Malaysian 2024 sylvatic strains and urban DENV2 strains. The exclusive presence of the shared mutations in both recent sylvatic human-infecting isolates and urban DENV2 strains, but not in earlier sylvatic viruses, raises intriguing possibilities about the underlying evolutionary mechanisms. One hypothesis is that these shared mutations may have arisen through recombination events between co-circulating urban and sylvatic DENV2 viruses, particularly during co-infections in human hosts. Although recombination in dengue viruses is considered rare, it is not unprecedented, especially under conditions, where cross transmission between ecological cycles occurs. Alternatively, the presence of these mutations may reflect adaptive convergence, whereby sylvatic strains, upon entering and replicating within the human host environment, acquire or retain mutations that confer a selective advantage similar to those already established in urban strains. This could include enhanced replication efficiency, immune evasion, or transmission potential in human hosts or *Aedes* mosquitoes. Importantly, the absence of these mutations in historical sylvatic DENV2 sequences suggests that this pattern is not a remnant of ancestral traits, but rather a more recent and dynamic interaction. Whether driven by recombination, convergent evolution, or a combination of both, this finding supports the idea that ecological spillover events can lead to genetic exchange or parallel adaptation, potentially facilitating the emergence of sylvatic strains with enhanced capacity for sustained human transmission. Further studies, including full-genome recombination analysis and fitness assessments, are needed to clarify whether these shared mutations result from inter-lineage recombination or adaptive evolution under selective pressures in the human–mosquito cycle. Future work is also expected to incorporate Bayesian time-scaled phylogenetic methods to estimate divergence times between sylvatic and urban dengue strains, which may reveal evolutionary patterns and zoonotic emergence timelines.

The identification of a divergent DENV3 Isolate (UDS93/1) raises the possibility that this strain may represent a previously undetected sylvatic DENV3 lineage. Although no molecular evidence has definitively confirmed the existence of sylvatic DENV3, its potential has long been speculated in literature. Neutralizing antibodies to DENV3 were previously detected in the seroconverted sentinel monkeys in Peninsular Malaysia, suggesting past exposure via a sylvatic route [19]. While current data are insufficient to conclusively classify UDS93/1 as sylvatic in origin, its genetic divergence from known urban DENV3 strains, combined with historical serological evidence, underscores the need for continued genomic surveillance and targeted ecological studies to investigate the existence and public health significance of sylvatic DENV3 in Malaysia.

The predominance of NS1-positive yet IgM/IgG-negative results reflect early phase infections, where seroconversion had not yet occurred at the time of sampling. This serological pattern aligns with the observed clinical data, where many patients were sampled within the first 5 days of illness. The high proportion of severe cases (86.4%) raises questions regarding potential pathogenicity differences between sylvatic and urban strains. Sylvatic strains which were known to be adapted to non-human primates may cause more aggressive pathology in humans due to incomplete host adaptation. Such mismatch may provoke heightened immune activation, resulting in vascular leakage, organ involvement, and shock. The fatal case, involving an 80-year-old male with positive NS1 but negative IgM and IgG, suggests a primary infection. Immunosenescence and comorbidities in the elderly may have contributed to a blunted immune response, rapid progression, and poor outcome. Unfortunately, sequencing failed for this sample, and a repeat was not possible, preventing genomic correlation with disease severity. Hence, this fatal case, although unsequenced, was categorized as a presumptive sylvatic DENV2 infection based on epidemiological context.

Emergence of sylvatic dengue in human population requires diagnostic accuracy to detect and differentiate it from the urban dengue serotypes. Antigen and antibody assays, such as the detection of NS1 antigen, IgM and IgG are used to identify acute dengue infections. NS1 is detectable in the serum from the onset of symptoms and before the appearance of antibodies, making it a valuable marker for early diagnosis. Based on the current study data, it is apparent that the NS1 assays such as rapid diagnostic test kits can detect sylvatic dengue as NS1-positive; however, they do not differentiate between sylvatic and urban strains. Even though the differences in the nucleotide sequence of sylvatic dengue viral genes are quite distinct from the DENV1-4 urban serotypes, this does not affect the NS1 protein conformation and secretion in infected human. In fact, a study found that NS1 antigen was detected in the sylvatic DENV2 sample at high concentration [11].

Real-time RT-PCR is commonly employed to detect DENV RNA in patient serum or plasma samples, yet most commercial kits are not designed to identify sylvatic lineages specifically. The extensive genetic variability across structural and non-structural genes of sylvatic dengue complicates primer design. Follow-up sequencing and phylogenetic analysis remain necessary for confirmation, though this is time-consuming and resource-intensive. There is an urgent need for diagnostic tools tailored to sylvatic dengue, enabling timely detection and outbreak mitigation. The conserved 5′ and 3′ UTRs offer promising targets, as evidenced by incidental detection of sylvatic strains using a broad-range 3′UTR-targeting assay in this study. Similarly, Cecilia et al. [20] reported high conservation of the 3’UTR within each serotype. Notably, our analysis revealed that the NS2a region was also found to be highly conserved within DENV2 serotype with no evidence of sylvatic-associated signature mutations. Therefore, the 3′UTR and NS2a regions are reliable targets for pan-dengue detection assays, with the potential to capture both endemic and sylvatic dengue strains. In addition, the development of multiplex PCR panels or specific primers tailored for sylvatic lineages would enhance diagnostic sensitivity and specificity, particularly in areas, where sylvatic and endemic dengue viruses co-circulate.

Uncontrolled deforestation and rural urbanization globally pose risk of widening the interface between humans and sylvatic transmission cycle. Global deforestation, primarily in developing nations, causes 13 million hectares of land loss annually, accounting for 31% of the world’s total forest cover [21]. Enhanced surveillance in forest-edge communities is essential, particularly, where human–wildlife–vector interactions occur. The detection of sylvatic dengue virus in human cases also underscores the critical relevance of the One Health framework, which recognizes the interconnectedness of human, animal, and environmental health. Integrated vector and wildlife surveillance, particularly in areas of human encroachment into forested regions, is crucial for early detection and risk mitigation.

This study has limitations that should be acknowledged. First, sequencing was unsuccessful in 13 samples, which limited the number of complete genomes available for analysis particularly to perform recombination detection which are necessary to validate some preliminary findings. Second, the study did not include environmental or entomological surveillance data, which could have provided additional context regarding transmission dynamics and factor influencing sylvatic dengue virus circulation. Future studies incorporating broader genomic sampling and integrated ecological data are necessary to build a more comprehensive understanding of sylvatic dengue emergence.

## Conclusion

Routine dengue surveillance at Malaysia’s national reference laboratory uncovered 22 human infections, including 8 confirmed sylvatic DENV2, one divergent DENV3 and 13 presumptive sylvatic dengue. Whole-genome sequencing confirmed the presence of a distinct Malaysian sylvatic DENV2 lineage, with mutations suggestive of recent adaptation, divergence, and possible genetic interaction with urban strains.

In light of deforestation activity and human encroachment into forested ecosystems, the likelihood of sylvatic dengue spillover is growing. These findings highlight the pressing need for improved diagnostic tools, enhanced surveillance in at-risk areas, and continuous genomic monitoring to ensure early detection and effective response to sylvatic dengue emergence.

## Data Availability

The genome sequences generated in this study are available in GISAID with accession numbers EPI_ISL_20063516, EPI_ISL_20063517, EPI_ISL_20063518, EPI_ISL_20063519, EPI_ISL_20063520, EPI_ISL_20063521, EPI_ISL_20063522, EPI_ISL_20063523 and EPI_ISL_20064972. Other data sets used during the present study are available from the corresponding author upon reasonable request.
